# Extraction of nettle (*Urtica dioica* L.) toxins under natural biting conditions

**DOI:** 10.1038/s41598-022-09916-0

**Published:** 2022-04-08

**Authors:** Ali Ammarellou

**Affiliations:** Research Institute of Modern Biological Techniques, University of Zanjan, 45371-38791 Zanjan, Iran

**Keywords:** Biochemistry, Biological techniques, Chemical biology, Physiology

## Abstract

A group of natural poisons from various animals, plants and microorganic sources can be extracted, produced and processed. Following ten years of field and laboratory research and studies, resulted from the creation of the first live collection of Iranian nettle ecotypes (LCINs) at the University of Zanjan, the feasibility of fresh and live extraction of nettle poison in pristine and untouched conditions was examined. In this study, the ability of tree tissues to absorb, hunt and sink nettle hairs, including styrofoam, nanofabric and sponge of the same length (15 cm) and same diameter (4 cm) having the same size of pores, was studied in four selected nettle ecotypes, including ecotypes of Mashhad, Mazandaran, Gilan and Zanjan provinces, Iran. For all four ecotypes on the three studied surfaces, the mean number of fully stuck and sunken needles, broken and sunken needles on the surface tissue, pores torn by plant needles and pores containing pale green liquid were counted and fully scrutinized. The results showed that sponges can be a suitable texture for hunting nettle hairs for extracting fresh and raw live venom of approximately 5 ml on a sponge source for 5 min. Based on GCMS analysis of total venom extraction resulting profile from the studied protocols had more than 10 compounds including some important sulfur containing such as: 2,2-dimethyl-propyl 2,2-dimethylpropanesulfinyl sulfone and 2-ethylthiolane, S,S-dioxide, etc. In this method, there is no need to remove the plant and stem. Its unique advantage is in continuous poison harvests during the 6-month growing season. Based on published research, this is the first report of live extraction of nettle medicinal poison.

## Introduction

The initial concept of biting in human mind and memory tends to be bitten more by animals such as snakes and scorpions and biting insects such as bees and mosquitoes. With the advancement of science and the discovery of new facts, human hatred has shifted from being bitten to positive concepts such as the extraction of poisonous drugs from snake and scorpion venom, as well as bee stings. Along with biting animals, biting plants are a familiar and tangible concept for local forests residents and rural people. The group of stinging plants, most widely known and distributed in the world, are stinging nettles (*Urtica dioica* L. Urticaceae)^1^. Stinging hairs of different kinds of nettles have fascinated botanists for centuries and have been studied extensively^1,2^. The method of urtication or rotation of patients' body on living and biting processes of nettle has a long history in the culture and the folklore of some countries in the world aiming at curing arthritic or paralytic limbs patients. The scientific behind this issue is to stimulate and accelerate blood flow to tissue resulting of nettle hairs^3^. The stinging hair is the first plant structure subject to microscopic study by Robert Hooke (1665)^4^. *Urtica dioica* (common nettle) and *U. urens* (burning nettle, lesser nettle, or dwarf nettle) are native to Europe and Eurasia, which grow wildly in mild and temperate climates, especially in forests and shady humid places in Europe, Asia, North Africa and North America^5–7^. The family name Urticaceae, generically known as *Urtica*, has its species’ name as *urens, which* are derived from the Latin verb *urere*, meaning “to burn,” a reference to the plant’s stinging hairs. The species name *dioica* comes from Greek for “two houses,”, which refers to the male and female flowers in separate plants (i.e., they are dioecious)^5,6^. The most famous nettle plant, which is sometimes called the queen or king of medicinal plants, is coined so due to its medicinal properties, such as antioxidant, antiplatelet, hypoglycemic and hypocholesterolemic properties^8,9^. Urtica dioica L. is rich in different ingredients depending on the part of the plant. The leaves are rich sources of terpenoids, carotenoids and fatty acids, as well as various essential amino acids, chlorophyll, vitamins, tannins, carbohydrates, sterols, polysaccharides, isolectins and minerals, while the roots contain oleanol acid, sterols and steryl glycosides^7–9^. In addition to medicinal and food industry uses^9,10^ of nettle, the analysis of the literature and published reports shows that attempts have been made to create biocomposites, reinforced with common nettle^10–12^ which is also regarded as a modification to improve compatibility between hydrophilic natural fiber and hydrophobic polymer matrix in the form of chemical treatment with NaOH^13^. Drying the plant limbs or cooking it with heat not only removes the biting property but it also destroys or changes the nature of the material in the nettle bite^14^. Hence, introducing a method for natural nettle venom collection from live plants (without plant harvesting) is necessary for industrial extraction of medicinal matter from this valuable plant. The main incentive of this research is to establish an efficient and practical method for collecting of nettles venom on natural bite conditions. Based on published research reports, this is the first study to scientifically focus on raw and natural nettle venom.

## Materials and methods

### Experimental site

The experimental site **was** located at latitude 36° 68′ N, longitude 48° 38′ E, and an altitude of 1579 m with a cold climate, a mean annual precipitation of 295 mm, and a mean annual temperature of 10 °C. The average minimum temperature in the coldest month **is** "Bahman to -7.5", and the average maximum temperature in the warmest month is "August, 32.1". During the year, the temperature drops below zero for 118 days, with January and February ranking first with 27 days. The wettest month is May with 52.5 mm, and the driest month is September with 3.5 mm.

The maximum recorded wind speed of 27 m per second was "97 km per hour". The prevailing wind in Zanjan in most months of the year was in the east, and the average wind speed of 3 m per second was "11 km per hour".

### Plant material

**Figure 1:**
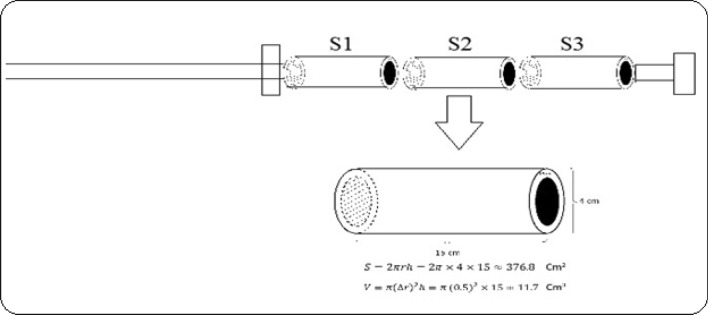
Schematic diagram of the structure and arrangement of circular surfaces collecting nettle venom. S1, S2 and S3 are three types of cylindrical circular surfaces: styrofoam, nanofabric and sponge.

The academic permission for collecting and researching medicinal plants was obtained from Head of Biotechnology, Department Research Institute of Modern Biological Techniques, University of Zanjan, Zanjan, Iran. The study complies with all relevant guidelines. All experiments were performed on 20 nettle ecotypes in the National Live Collection of Iranian Nettle (LCINs), located at the Research Institute of Modern Biological Techniques, University of Zanjan, Zanjan, Iran. A total of 4 nettle ecotypes (4 different provinces of Iran), similar in age and growth morphological conditions, were randomly selected and further used to collect nettle bites and venom. These nettle ecotypes were collected from different nettle habitats in Iran from different provinces in Iran, including Mazandaran, Gilan, Mashhad and Zanjan. These collections were cultivated in both greenhouse and field conditions and were raised and maintained (2013–2022) (Fig. 1).

### Morphology study of explants

All stages of hair penetration in to 3 different surfaces were analyzed during the research process, and sample stems, leaves and hairs were documented with a stereoscopic microscope (Nikon SMZ1, Tokyo, Japan). Photographs were taken with an Olympus microscope (Olympus CX31, Tokyo, Japan) at 40 and 400 magnifications.

### Collection of poison

Three small porous circular plates with a length of 15 cm and a diameter of 4 cm in three types of styrofoam, nanofabric and sponge of the same length (15 cm) and same diameter (4 cm) were embedded on a light rod and so that they could be rotated on the rod in accordance with the rod axis.

### GCMS analysis

GC–MS analysis of the extract was performed via an Agilent 7890B (USA) gas chromatograph equipped with an HP5-MS (5% phenyl/95% dimethylpolysiloxane) capillary column (60 m × 0.25 mm 1D × 0.25 μm). This was coupled with a 5977A mass spectrometer in the electron impact (EI) ionization mode with an ionizing energy of 70 eV. . Helium gas (99.999%) was used as the carrier gas at a constant flow rate of 1 ml/min, and an injection volume of 1 μl was employed (split ratio of 1:20 ) under following conditions:injector temperature 280 °C, ion-source temperature 230 °C and MS transfer line of 300 $^\circ$C. The oven temperature was programmed from 70 °C (isothermal for 5 min.), with an increase from 15 °C/min to 200 °C and then 8 °C/min going up to 280 °C, and held for 10 min. A full scan interval of 0.5 s and fragments from 45 to 600 amu (Da) was programmed in the mass detector part^15^. A relative % amount of each component was calculated by comparing its average peak area to the total peak areas. The interpretation on mass spectrum GC–MS was conducted using the database of National Institute Standard and Technology (NIST) and Wiley Registry of Mass Spectral Data, 6th Edition (Wiley Interscience, New York) with more than 140,000 patterns.

### Statistical analysis

The experimental design was employed with 4 × 3 factorial treatments based on a randomized complete block design (RCBD) having three replications. The ecotype in 4 levels (Mashhad, Mazandaran, Gilan and Zanjan) and the circular plate in three levels of Styrofoam, Nanofabric and Sponge were used in this study. The analysis of variance (ANOVA) and Duncan’s multiple range test (DMRT) were used to analyze the data, which was performed by SAS software (SAS Institute, 2003).

## Results

**Figure 2:**
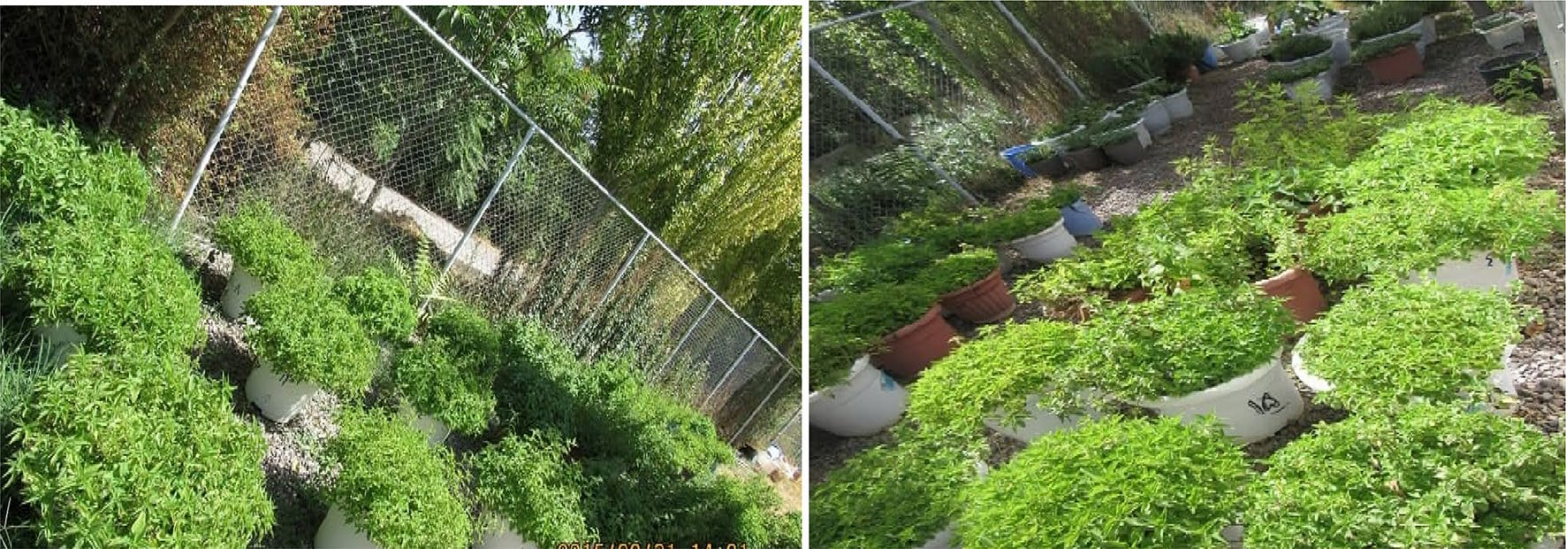
The Live Collection of Iranian Nettle (LCIN) is located at the Research Institute of Modern Biological Techniques, University of Zanjan, Zanjan, Iran, at latitude 36° 68′ N, longitude 48° 38′ E.

**Figure 3:**
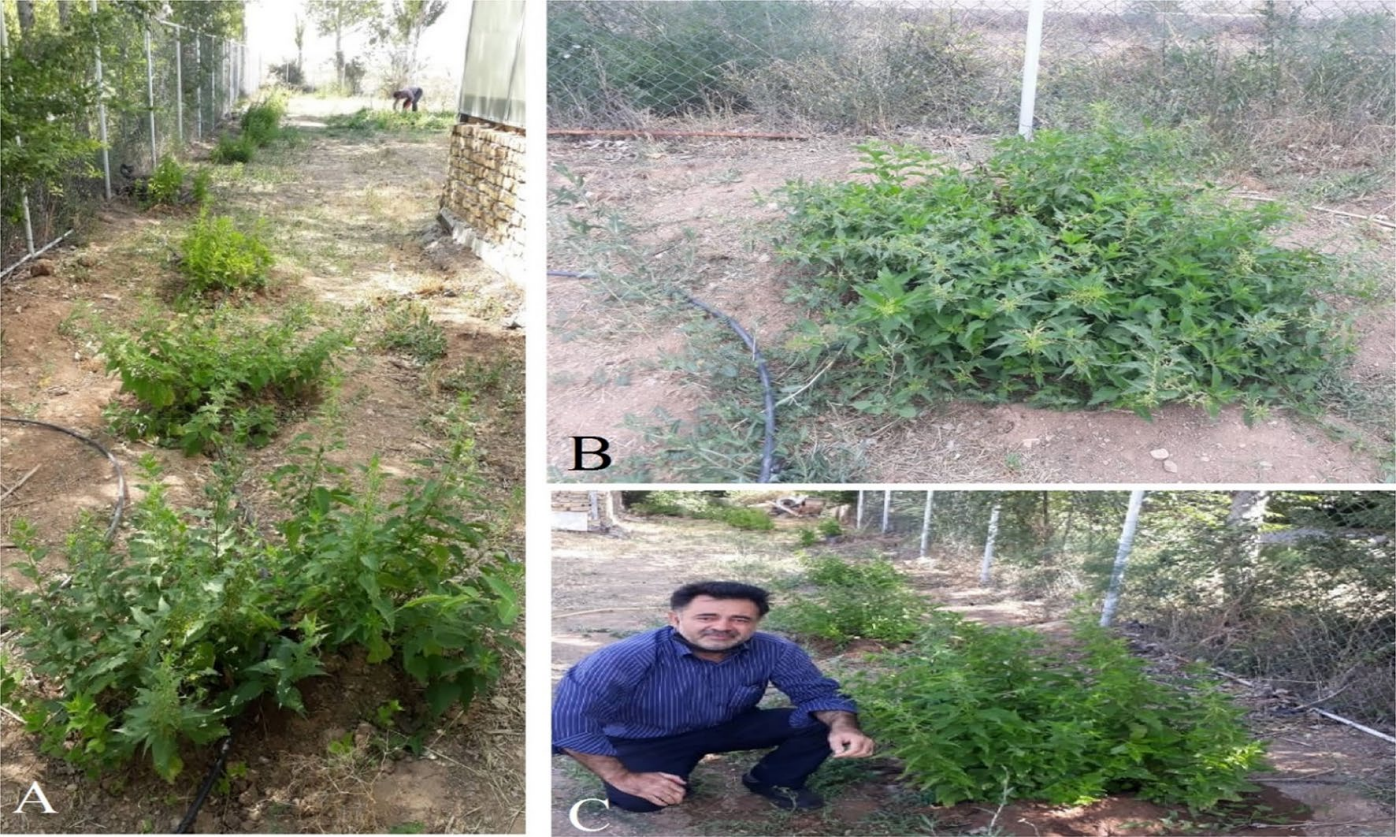
Field experimental studies on Nettles (**A**), a developed 2-year nettle plant (**B**) and Ali Ammarellou, the researcher and author on the Live Collection of Iranian Nettles (LCINs). Shrubs are planted in the field with similar soil conditions at intervals of three meters having irrigation system in drops.

**Figure 4:**
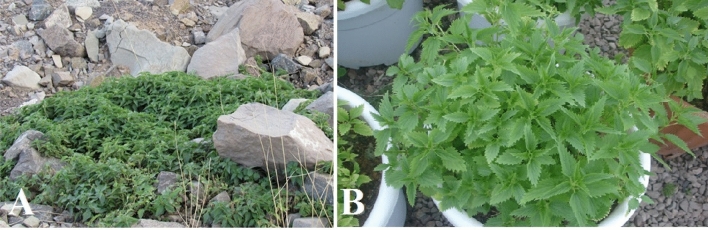
The wild habitat of a mountainous nettle (**A**) and the same plant under pot growth conditions (**B**).

**Figure 5:**
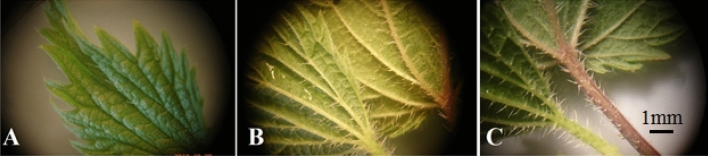
Comparison of stinging hairs frequently on the leaf surface (**A**), behind close-up of leaves (**B**) and nettle leaf tail.

**Figure 6:**
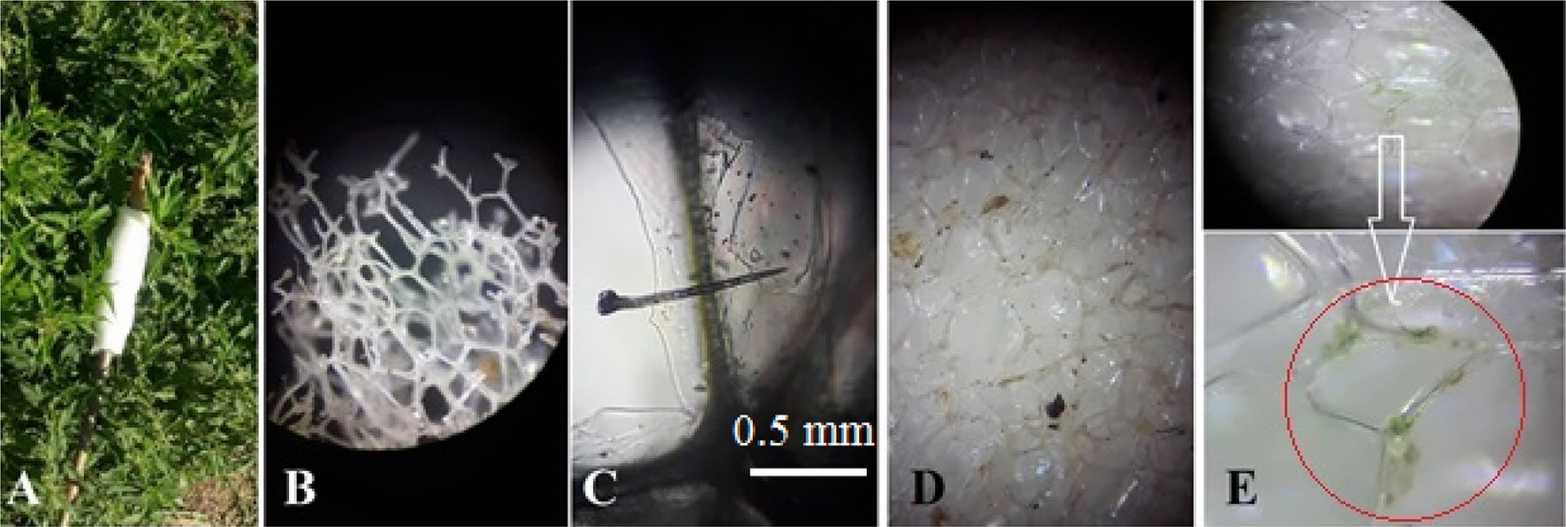
The use of an innovative rod containing the hunting surfaces )**A**) Small nettle hairs, as well as the final liquid of the hairs (**B**,**C**) sitting on the porous surfaces of the adsorbent and meticulously collected (**D**). The greenish-white liquid that sitting on the pore wall of the studied surfaces having the same biting content and also having the active ingredient of the nettle plant marked in a red circle (**E**).

**Figure 7:**
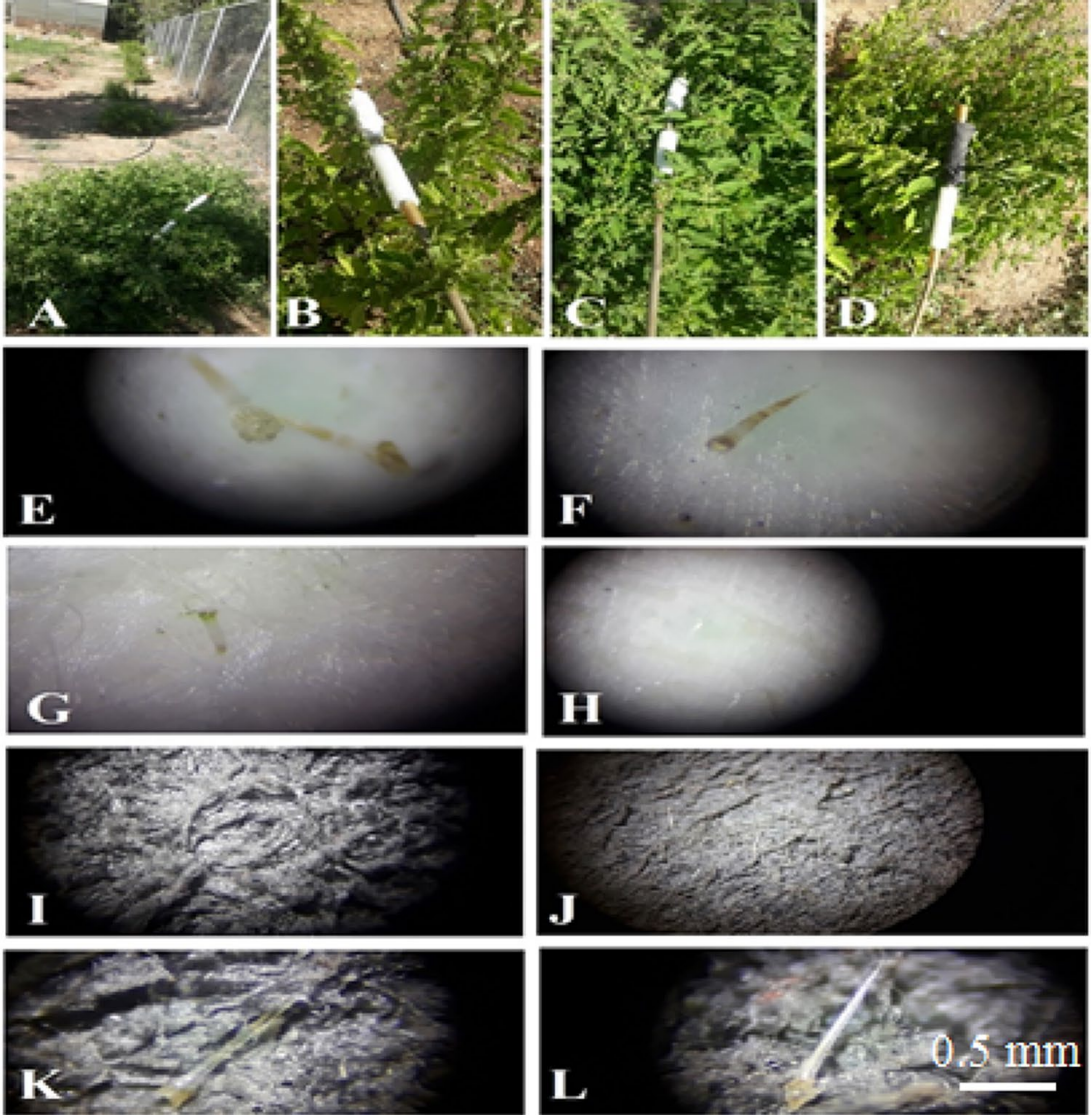
Repeated scientific experiments with different levels of rolling and circular surfaces of different dimensions and sizes (**A**–**D**), The immersion of hairs and nettle needles in the soft depth of circular surfaces and strong structure of needles (**E**–**G**) special ornaments at the beginning of the crack (**E**). A faint liquid seen on the surface of the Styrofoam (**H**). The surface of the nanofabric before the bite (**I**) and the same fabric after the bite (**J**–**L**) are shown.

**Figure 8:**
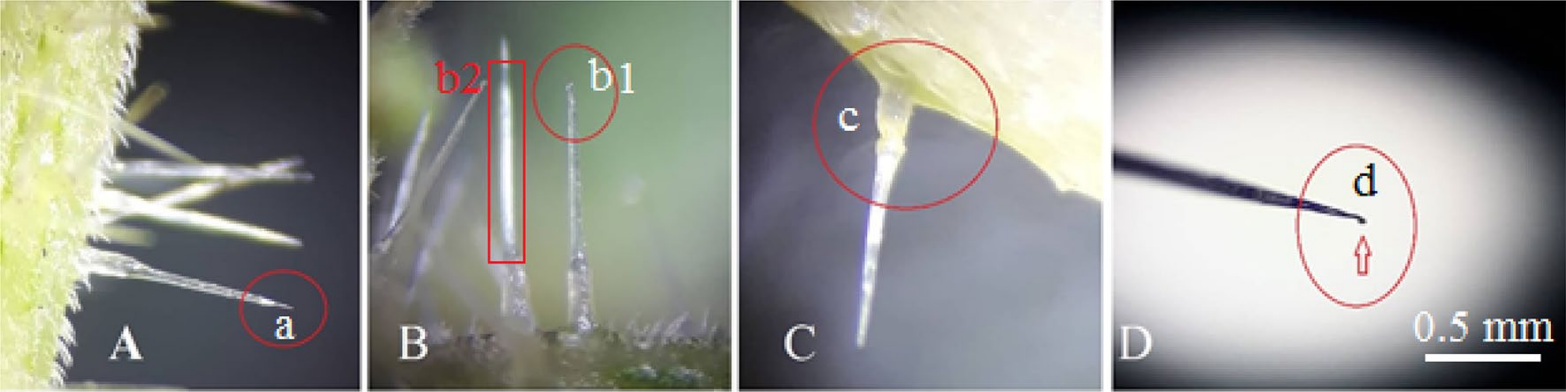
Morphological details of stinging hairs and their distribution on the surface of the tissue. Most of them have three parts: a poison tank (c), a needle or injection tube (b2), and a special brittle tip marked with a red circle (b1& d).

**Figure 9:**
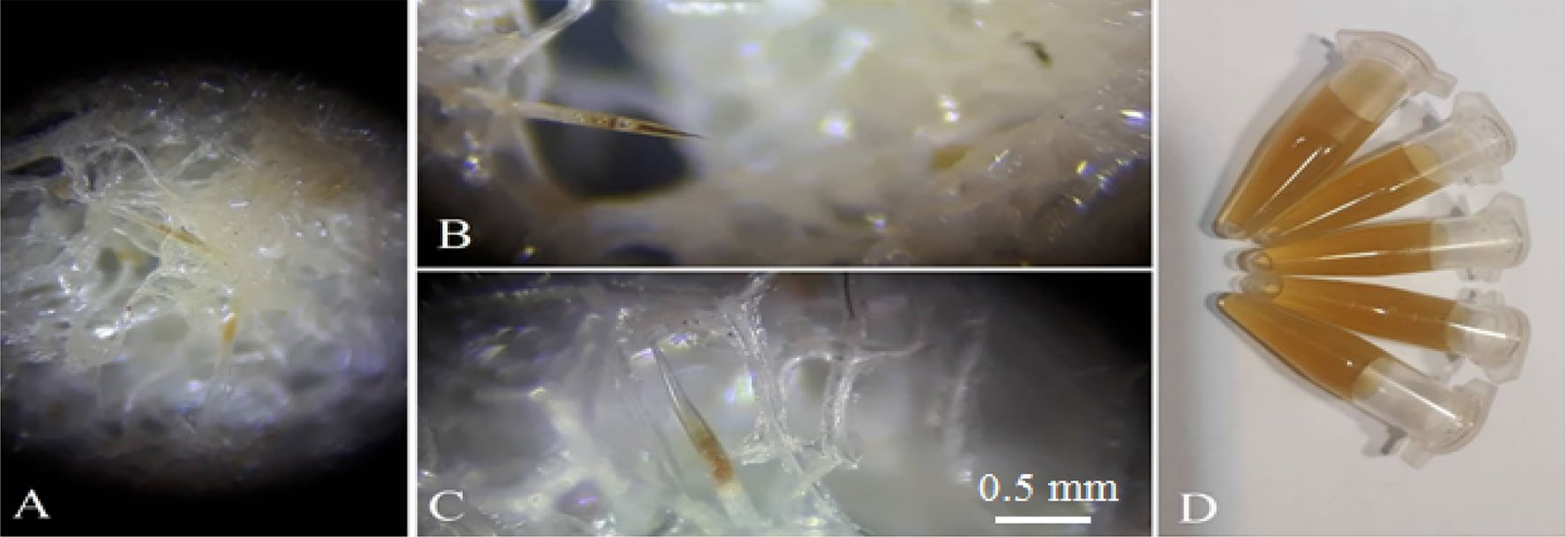
Injectable fluid fixed in needles penetrated into the studied surface with dark yellow color (**A**–**C**) and venom extract collected from a nettle garden (**D**).

The photos below show the live ecotypes of nettles in the Live Collection of Iranian Nettles (LCINs) located at the University of Zanjan with more than 50 nettle ecotypes. These plant specimens were studied under both greenhouse and field living conditions. This collection has been established since 2013 (Figs. 2, 3). Using a one-and-a-half meter innovative rod in which the three studied surfaces, including Styrofoam, Nanofabric and Sponge as circular and rolling surfaces, were randomly installed and designed in the same size and dimensions for two minutes, which were completely and slowly performed on the leaves and stems (Fig. 1). Each of the rotating cylindrical surfaces had a side area of 376.8 cm^2^ and a final volume of 11.7 cm^3^, but the volume of the sponge mass had to be reduced from the maximum total amount. Ecotypes have just been contacted. Immediately after contact, the surfaces used were immediately placed under a stereomicroscope, which was located in the same place, and consequently the desired surfaces were carefully evaluated and photographed. For all four ecotypes on the three surfaces studied, the mean number of fully stuck and sunken needles, broken and sunken needles on the surface tissue, pores torn by plant needles and pores containing pale green liquid were counted and noted (Figs. 4, 5, 6, 7, 8, 9). The results of surveys and data analysis are given in Table 1.

**Table 1:** Comparison of means for surface effects on four markers of nettle venom collection.

Treatments		Mean value of fully stuck needles (per cm^2^)	Mean value of broken and sunken needles (per cm^2^)	Mean of pores torn by plant needles (%)	Mean value of pores containing pale green liquid (%)
Mazandaran	Styrofoam	10.4^c^	9.3^c^	50^b^	20.4^c^
	Nanofabric	18.2^b^	23.1^b^	5^c^	60.9^b^
	Sponge	50.5^a^	40.2^a^	95^a^	70.3^a^
Gilan	Styrofoam	10.1^c^	8.8^c^	48.6^b^	19.7^c^
	Nanofabric	19.2^b^	22.3^b^	5.3^c^	62^b^
	Sponge	55.3^a^	41.7^a^	94.1^a^	71.7^a^
Mashhad	Styrofoam	9.7^c^	9.3^c^	45^b^	18.9^c^
	Nanofabric	19.5^b^	23^b^	4.7^c^	63.1^b^
	Sponge	56.1^a^	43.6^a^	95.4^a^	72.6^a^
Zanjan	Styrofoam	9.8^c^	10^c^	51^b^	21.2^c^
	Nanofabric	17^b^	18.4^b^	6^c^	62.8^b^
	Sponge	51.2^a^	42^a^	93^a^	73.4^a^

Treatments with at least one letter in common showed no significant difference.

**Figure 10:**
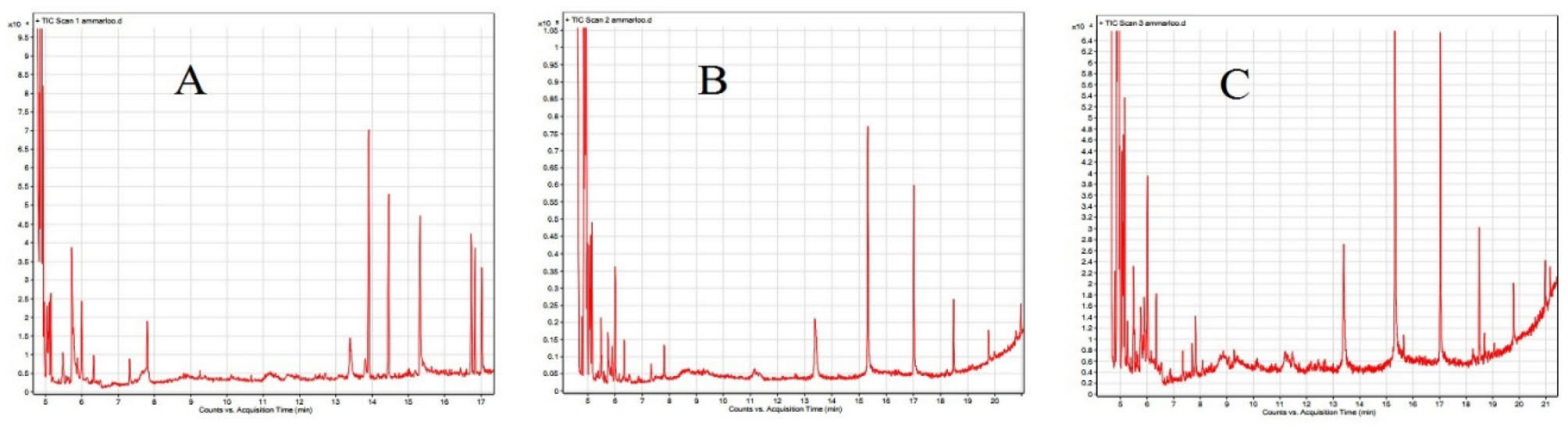
GCMS chromatograms of nettle hair raw extracts with N-hexane + methanol in 3 repeats (**A**–**C**). Because most of the experimental peaks were observed up to 17 min, no other important peaks were observed after 17–20 min.

Based on Fig. 10. Total venom extraction resulting profile from the studied protocols had more than 10 compounds including some important sulfur containing such as: 2,2-dimethyl-propyl 2,2-dimethylpropanesulfinyl sulfone and 2-ethylthiolane, S,S-dioxide, etc. The main components were presented in Table 2.

**Table 2:** Some of the properties of identified compounds in nettle venom based on GC–MS analysis.

Compound name	RT^a^ (min)	MW^b^	Quantitation ion (m/z)	RI^c^ (retention index)	Identification method^d^
Valeric anhydride	4.85	186	85	1283	1,2
Ethyl thiolane, S,S dioxide	4.97	148	56	-	2
2,2-dimethyl-propyl 2,2-dimethyl-propanesulfinyl sulfone	5.15	254	71	1824	1,2
1,2-dimethyl Cyclopentane	5.11	130	72	874	1,2
Cyclobutanone, 2-(1,1-dimethylethyl)-	5.48	126	69	926	1,2
4H-1-Benzopyran-2-carboxylic acid, 8-amino-7-hydroxy-4-oxo-, ethyl ester	25.72	249	249	2297	1,2
Glycine, 2-cyclohexyl-N-octyloxycarbonyl-, isohexyl ester	29.55	397	268	2650	1,2

^a^Retention time.

^b^Molecular weight.

^c^Calculated Retention indices based on C_6_-C_24_ n-alkanes on the HP-5MS: 5% phenylmethylpolysiloxane (SE-54, DB-5 ms, CPSi18, etc.).

^d^1-Retention indices; 2-NIST & Wiley libraries.

The results showed that sponges can be a suitable texture for hunting nettle hairs for extracting fresh and raw venom. The production of graphs in three replications, as described in Fig. 10, shows the scientific and executive success of the presented protocol. Based on published research, this is the first report of live extraction of nettle medicinal poison.

## Conclusion

Based on the statistical analysis of the data, the cylindrical plate of the sponge has more communication and connection power and hunting of fine nettle hairs than other levels, and it is suggested and introduced for additional experiments on the extraction and purification of nettle venom. Another advantage of this porous texture (sponge) is that it easily penetrates the water and easily loses the absorbed water. This convenience and ease of water loss in nanofabric is much less, and basically not much water is absorbed in the foam. This valuable physical property of the sponge is better for extracting the active ingredients that are involved and accumulate in the pores of the tissue with a suitable solvent such as water.
